# Clinical, pathologic and molecular findings in 2 Rottweiler littermates with appendicular osteosarcoma

**DOI:** 10.21203/rs.3.rs-4223759/v1

**Published:** 2024-04-11

**Authors:** Kate I. Silver, Joshua D. Mannheimer, Corey Saba, William P. D. Hendricks, Guannan Wang, Kenneth Day, Manisha Warrier, Jessica A. Beck, Christina Mazcko, Amy K. LeBlanc

**Affiliations:** National Cancer Institute, NIH; National Cancer Institute, NIH; University of Georgia; Vidium Animal Health, A Subsidiary of The Translational Genomics Research Institute (TGen); Vidium Animal Health, A Subsidiary of The Translational Genomics Research Institute (TGen); Vidium Animal Health, A Subsidiary of The Translational Genomics Research Institute (TGen); Vidium Animal Health, A Subsidiary of The Translational Genomics Research Institute (TGen); National Cancer Institute, NIH; National Cancer Institute, NIH; National Cancer Institute, NIH

**Keywords:** Osteosarcoma, canine, Rottweiler, genomics

## Abstract

Appendicular osteosarcoma was diagnosed and treated in a pair of littermate Rottweiler dogs, resulting in distinctly different clinical outcomes despite similar therapy within the context of a prospective, randomized clinical trial (NCI-COTC021/022). Histopathology, immunohistochemistry, mRNA sequencing, and targeted DNA hotspot sequencing techniques were applied to both dogs’ tumors to define factors that could underpin their differential response to treatment. We describe the comparison of their clinical, histologic and molecular features, as well as those from a companion cohort of Rottweiler dogs, providing new insight into potential prognostic biomarkers for canine osteosarcoma.

## Introduction

Osteosarcoma is the most common form of primary bone cancer found in both humans and canines.^1,2^ Human and canine osteosarcomas share many clinical, histologic and molecular features, such as low overall tumor mutational burden, frequent somatic translocations and mutations, and tumor site.^3–5^ Osteosarcoma is thought to arise from primitive bone cells and often produces an osteoid-containing matrix.^6–8^ Osteosarcomas generally develop in the appendicular skeleton of both species, typically in the distal femur or proximal tibia in humans, and the distal radius and proximal humerus in dogs.^9–11^ Osteosarcoma is highly aggressive and tends to rapidly metastasize to the lungs, although disease progression to soft tissue, bone, and other internal organs, has been reported as well. Unfortunately, the survival rates for humans and their canine counterparts are low compared to other cancers due to the metastatic nature of osteosarcoma and the stagnation in development of effective novel therapeutic treatments over the last several decades.^12–15^

Despite these similarities, the disease incidence for humans drastically differs from canines; over 10,000 mainly large breed dogs are diagnosed with osteosarcoma per year, while less than 1,000 human cases are reported annually in the US.^2–3^ In addition, osteosarcomas disproportionately develop in children and adolescents between 10 and 14 years old, pointing to periods of rapid skeletal growth as a risk factor in tumor development.^16–17^ However, canines diagnosed with osteosarcoma are typically between 7 and 10 years old and fall within the adult or geriatric age group.^11^ Due to the rarity of osteosarcoma in children and the high frequency of spontaneous canine cases, the pet dog has become an invaluable asset for researching effective cancer therapies for humans with cross-species applications.^18–20^

Certain canine breeds reportedly possess a higher risk of developing osteosarcoma than others, such as the Irish Wolfhound, Scottish Deerhound, and Rottweiler.^21–24^ While the literature conflicts over which dog breeds are most predisposed to the disease, one study found that Rottweilers have a higher osteosarcoma odds ratio than any other breed.^24^ Rottweilers are also one of three breeds included in a large osteosarcoma Genome Wide Association Study (GWAS), making them an ideal candidate for uncovering genomic biomarkers in dogs with translational value for humans.^3,25^ In addition, Rottweilers may perform worse than other dog breeds despite receiving the same cancer treatment, though this evidence is observational.^21^

The Comparative Oncology Program (COP) within the intramural research program of the National Institutes of Health - National Cancer Institute launched the Comparative Oncology Trial Consortium (COTC) in 2003 to utilize the pet dog as a translational model for novel cancer treatments.^26^ Under the COTC infrastructure and with support of the Morris Animal Foundation, a 2-armed prospective, randomized trial was conducted in canine osteosarcoma patients, COTC021/22. Dogs enrolled in this trial received either Standard of Care (SOC) therapy, or SOC + adjuvant sirolimus (SOC + S) therapy.^27^ The trial enrolled 324 dogs, 18 of which were Rottweilers. Of this cohort, two Rottweiler dogs, patient ID numbers 1410 (spayed female) and 1411 (castrated male), were littermates and were both diagnosed with osteoblastic osteosarcomas within 3 months of each other. Both dogs, aged 6 at the time of osteosarcoma diagnosis, were raised in the same household and received treatment from the same veterinary teaching hospital, yet had vastly different clinical outcomes. The purpose of this case report is to review the clinical, histopathologic, and genomic features of these dogs, and attempt to define characteristics that underlie the shared incidence of disease as well as the disparate outcomes of canine siblings affected by osteosarcoma.

## Methods

The clinical trial structure, methods and results for COTC021/022 have been reported elsewhere.^27^ Specific to the Rottweiler cohort, medical records and clinical data provided for dogs 1410 and 1411, along with the 16 other Rottweiler dogs represented in the COTC021/022 trial were reviewed, along with imaging studies, histopathology, and transcriptional profiles of primary tumor tissues. Additionally, mRNAseq data from the dogs’ primary tumors, which was generated in a prior study^28^, was incorporated and included in the analysis presented here. DNA was isolated from tumors and matched normal tissue samples from dogs 1410 and 1411 and subjected to profiling using a cancer genomic diagnostic assay (SearchLight DNA; Vidium Animal Health). Pharmacokinetic profiling of sirolimus exposure in dog 1411 collected from the COTC021/022 trial was also reviewed.

### Histopathology

Primary tumor biopsies were obtained prior to treatment at the time of limb amputation, and were evaluated by anatomic veterinary pathologists at participating COTC institutions (https://ccr.cancer.gov/comparative-oncology-program/consortium). Surgical histology reports of the primary tumor were reviewed by the COP investigative pathologist (JAB). Tumor sections were labeled for CD204 by the Animal Health Diagnostic Center at Cornell University (Cosmo Bio, KAL-KT022; 1:500).

### Nucleic acid isolation and sequencing

DNA and RNA were isolated from canine frozen tumor and normal tissue in RNAlater using Qiagen Allprep DNA/RNA Mini Kit (Cat#80204). The total RNA quality and quantity was assessed using Nanodrop 8000 (Thermofisher) and Agilent 4200 Tapestation with RNA Screen Tape (Cat# 5067–5576) and RNA Screen Tape sample Buffer (Cat#5067–5577). All samples forwarded for mRNA sequencing had a RIN > 8 and a total RNA quantity > 100 ng. DNA quantity and quality were assessed using the Qubit Fluorometer 2.0 with the Qubit dsDNA BR assay (ThermoFisher Scientific) and the TapeStation genomic DNA assay (Agilent Technologies). Samples with a DIN > 3 and with > 50 ng total DNA were utilized for DNA sequencing.

### Library preparation and mRNA sequencing

Between 100ng to 1μg of total RNA was used as the input for the mRNA sequencing libraries. Libraries were generated using the TruSeq Stranded mRNA library kit (Illumina) according the to the manufacturers protocol. The libraries were pooled and sequenced on NovaSeq S1 using a 2×150 cycle kit. The HiSeq Real Time Analysis software (RTA v.3.4.4) was used for processing raw data files. The Illumina bcl2fastq2.17 was used to demultiplex and convert binary base calls and qualities to fastq format. The samples had 44 to 61 million pass filter reads with more than 91% of bases above the quality score of Q30. Reads of the samples were trimmed for adapters and low-quality bases using Cutadapt. The trimmed reads were mapped to the CanFam4 reference genome (GSD_1.0 from NCBI)^29^ using STAR aligner (version 2.7.0f) with two-pass alignment option. RSEM (version 1.3.1) was used for gene and transcript quantification based on the CanFam4 GTF file. The average mapping rate of all samples was 83% with unique alignment above 66%. There were 13.13–26.26% unmapped reads. The mapping statistics were calculated using Picard software. The samples had between 0.01–0.76% ribosomal bases. Percent coding bases were between 58–71%. Percent UTR bases were 10–16%, and mRNA bases were between 75–82% for all the samples. Library complexity was measured in terms of unique fragments in the mapped reads using Picard’s MarkDuplicate utility. The samples had 48–78% non-duplicate reads.

### mRNA sequencing data analysis

The COTC021/022 trials enrolled a total of 324 dogs with appendicular osteosarcoma. The DOG^2^ cohort consists of a subset of 186 canine osteosarcoma patients for which mRNAseq data from their treatment-naïve primary tumors is available. Eleven of the n = 186 dogs were of Rottweiler breed, including dogs 1410 (poor responder) and 1411 (elite responder). Of these 11 Rottweilers, 4 dogs were assigned to a group of “elite” responders (patient IDs 1411, 0608, 0511, 0301) with a median disease-free interval (DFI) of 453 days (range: 210–859 days) and a median overall survival (OS) of 826 days (range: 634–909 days). Six dogs were assigned to a group of “poor” responders (Patient IDs 0402, 0712, 1022, 1103, 1409, 1410) with a median DFI of 87 days (range: 55–126 days) and a median OS of 142 days (range: 75–194 days). One Rottweiler dog, 0518, was not included in this analysis because it was taken off study at 12 days post-operatively as the owner elected not to pursue further therapy.

Differential expression analysis from the available mRNAseq datasets described above was performed using the R (version 4.03) package DESeq2 (version 1.30.1).^30^ Confounding covariates for batch effects and sex were accounted for in the DESeq2 model. A p-value cutoff was determined using Independent Hypothesis Weighting (IHW) with significance level of 0.05 using the IHW (version 1.18.0) R package.^31^ Hierarchical clustering was performed with Ward’s method using the function “clustermap” function from seaborn (version 0.10.1) Python (version 3.8.3) package using the Log 2 DESeq normalized counts per million (CPM) expression data.

Based on our previous work with the DOG^2^ mRNAseq cohort, a 27 gene signature (GS-1) was shown to cluster canine primary tumors into two groups based on their relative expression of immune-related genes.^28^ Using this signature, the expression profiles were sorted by group (Immune-high, Immune-low, Rottweiler breed) and displayed in a heatmap composed using the seaborn (version 0.10.1) Python (version 3.8.3) package.

### DNA sequencing and data analysis

A pan-cancer genomic sequencing panel was applied to DNA extracted from treatment-naïve tumor and matched normal tissue samples collected from both dogs at the time of limb amputation. Tumor and matched normal samples underwent sequencing of targeted genomic regions using a proprietary panel of hybridization-based capture probes targeting 120 cancer genes as previously described (SearchLight DNA; Vidium Animal Health).^32–34^

Data was analyzed using a custom tumor-only genomics pipeline for the identification of SNVs, CNVs, and ITDs. The first step involved using Trimmomatic (v0.36)^35^ to remove adapter sequences, low-quality bases, and other artifacts, and to generate FASTQ quality control metrics. Trimmed paired-end reads were then aligned to the canine reference genome, CanFam v3.1.99^36^, using BWA-mem (v0.7.17)^37^. Consensus SNV/indel calls from Mutect2 (GATK-4.1.4.0)^38^ and Pisces (v5.2.5.20)^39^ were determined, and calls occurring at variant allele frequencies ≥ 3% were functionally annotated using SnpEff (v4.3)^40^ to determine the effects of the variants on the encoded protein. The Ensembl Variant Effect Predictor (VEP)^41^ was then utilized to determine the impacts of amino acid substitutions, incorporating SIFT annotation to assess the potential functional consequences. SIFT scores range from 0 to 1, with a lower score indicating a higher likelihood of being damaging to protein function. Substitutions with SIFT scores < 0.05 were considered high-impact (‘HIGH’), while substitutions with scores ≥ 0.05 and < 0.5 were considered moderate impact (‘MODERATE’). Substitutions with SIFT scores ≥ 0.5 were considered tolerated and marked as ‘BENIGN’.

Variants with a predicted impact of “HIGH” or “MODERATE” were subjected to additional filtering to exclude likely germline variants based on their presence in the European Variant Archive (EVA)^42^ with a population allele frequency (AF) of ≥ 1% in studies comprising at least 10 dogs in each cohort. In addition to these filtering steps, we also annotated potential biomarker associations using our Precision Oncology Knowledgebase (Vidium Animal Health). This approach enables the identification of mutation biomarkers that have been described in human or canine cancers in published literature. Mutations identified in both the constitutional samples and the matched tumor samples were considered in downstream analysis. Mutations were considered somatic if present only in the tumor sample and germline if present in constitutional and tumor DNA.

Manta (v1.6)^43^ was used for ITD calling of KIT and FLT3 genes, and CNVkit (v0.9.6)^44^ for CNV calls. A two-copy loss in tumor suppressor genes and a six-copy gain in oncogenes were assumed to have a significant impact on function. For copy number events, gains in autosomal oncogenes were retained if the confidence interval (CI) lower bound > 0.368, and losses in autosomal tumor suppressors were retained if the CI upper bound < −0.238. For genes on the sex chromosomes in males, a true gain was considered to have a CI lower bound > −0.7, and a true loss was any event with a CI lower bound < −1.3.

FASTQ files were generated and aligned to the canine reference genome, CanFam3.147. The primary analysis pipeline was automated to generate single-nucleotide variants (SNV), copy number variants (CNV), and internal tandem duplications (ITD), using the DNAnexus cloud-based computing platform (DNAnexus Inc.). Based on log2 fold change and tumor content, copy number gains or losses were inferred as single or multiple. For both 1410 and 1411, the Spearman correlation was calculated between the CNV variation reported from the SearchLight panel and scaled log 2 transformed DESeq normalized gene expression data. Additionally, expression values for genes exhibiting CNV events in either 1410 (poor) and 1411 (elite) were combined and the Spearman correlation between CNV variation and expression was calculated.

## Results

### Clinical findings

**Figure 1:**
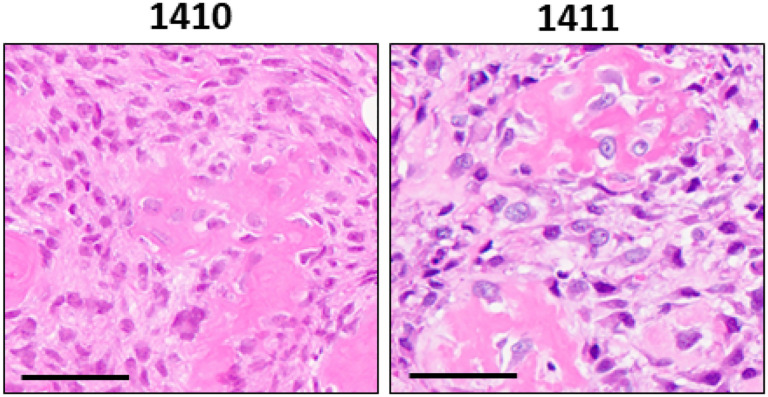
Primary tumor histopathology of dog 1410 and 1411. Representative images of tumor tissue from dogs 1410 and 1411 stained with hematoxylin & eosin (H&E). Scale bar = 50 μm.

**Figure 2:**
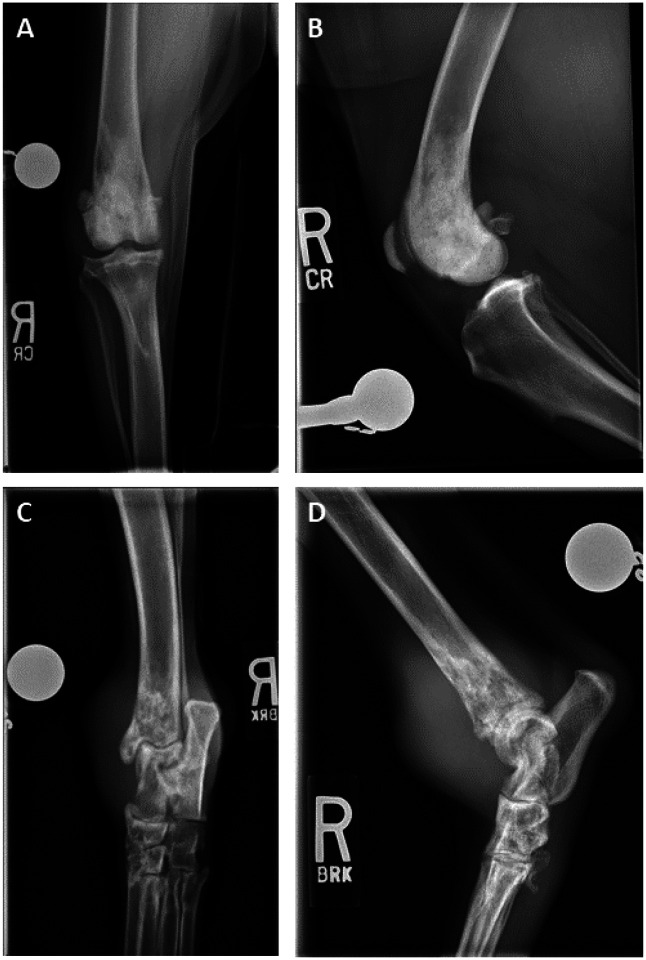
Radiographic images from dog 1410 and 1411. Right hindlimb radiographs of stifle joint of dog 1410 (poor outcome) taken at the time of diagnosis (panel A: anterior-posterior projection, B: lateral projection). In the distal metaphysis of the right femur, there is a mild moth-eaten bone lysis and marked sclerosis extending into the distal diaphysis and epiphysis. Radiographic images from dog 1411 (elite outcome), also from the right hindlimb but highlighting the tarsal joint (panel C: anterior-posterior projection, panel D: lateral projection). At the distal metaphysis and epiphysis of the tibia there is moth eaten lysis. The cranial and caudal margins of the cortex are smooth but thinned. Within the mid diaphysis, the medullary cavity has a mottled appearance. Circumferentially to the tarsus and within the tibiotarsal joint, there is a severe (more severe dorsomedially) amount of soft tissue swelling.

Dog 1410 (poor) presented with a 3×3×5 cm mass on the right distal femur and had a disease-free interval (DFI) of 62 days. However, her littermate, dog 1411(elite) presented with an 8×7×7 cm mass on the right distal tibia and had a DFI of 859 days, surviving nearly 13 times longer than their sibling. Both dogs were diagnosed with osteoblastic osteosarcomas (Fig. 1). Neither had evidence of lymphatic or vascular invasion in evaluated sections. A higher mitotic index was reported for 1411(elite) (20 vs. 8 in ten 400x fields). Serum Alkaline Phosphatase (ALP) levels for 1410 (poor) were normal but elevated for 1411; however, 1411 had a preexisting diagnosis of idiopathic epilepsy and history of phenobarbital treatment, which may have been responsible for the baseline elevation in liver-associated ALP. Radiographic findings prior to surgical limb amputation for both dogs were consistent with appendicular osteosarcoma (Fig. 2). Metastatic progression was documented in both dogs during the study period. 1411 (elite) had metastasis to the right distal femur, detected at 122 weeks (859 days) post-amputation, and 1410 (poor) to the lungs detected at 9 weeks (63 days) post-amputation. Neither patient underwent a post-mortem examination.

There were 16 other Rottweilers with osteosarcoma enrolled in COTC021/022. These dogs were not first-degree relatives with 1410 and 1411 and were included in this case report as a basis of comparison to the sibling Rottweilers and to the overall COTC021/022 cohort. The non-sibling Rottweiler cohort comprised 5 females and 11 males with a mean age of 7.1 years. The average Rottweiler weight was 42.4 kg for females and 50.8 kg for males. Serum ALP values varied equally within the non-sibling Rottweilers with 50% reporting elevated levels and 50% reporting normal levels. The majority of the non-sibling cohort (69%) was treated with SOC consisting of surgical limb amputation and carboplatin chemotherapy, while 31% also received adjuvant sirolimus (SOC + S). Most Rottweilers (75%) received 4 doses of carboplatin; the remaining dogs received fewer doses before exiting the study. The most common reason dogs were taken off study was disease progression (94%) while the most common method of death was euthanasia (81%). At the trial’s conclusion, all but one Rottweiler had documented metastatic disease and 44% had metastases in multiple locations. The most common sites of metastases were lung (81%), bone (31%), and kidney (31%). The median disease-free interval (DFI) for all non-sibling Rottweilers was 143 days and did not vary significantly from 1411 and 1410 (p = 0.6) or the overall median DFI for the COTC 021/022 trial (Standard of Care median DFI, 180 days; Standard of Care + sirolimus median DFI, 204 days).^27^

### Sirolimus pharmacokinetics

1410 and 1411 were randomly assigned to one of two treatment arms as part of the COTC021/22 trial: SOC or SOC + S. Dog 1411 (elite) completed 4 doses of carboplatin and 4 doses of sirolimus after limb amputation, whereas dog 1410 (poor) received the SOC treatment after amputation and only 2 doses of carboplatin before removal from study due to disease progression. Ultimately, the results of COTC021/22 found that there was no significant difference between SOC + S and SOC with respect to DFI and overall survival time.^27^ This could be due to the high variability in oral drug absorption and bioavailability of sirolimus and is further supported by 1411’s pharmacokinetic summary (**Supplemental Table 1**), which demonstrated an estimated trough level of sirolimus far below 10 ng/ml, which is the exposure threshold for the drug thought to exert therapeutic efficacy in sarcoma.^45^ Thus, although its contribution cannot be ruled out, the treatment type 1411 received was thought not to be the primary determinant of their extended survival time compared to 1410.

### Genomic profiles

**Figure 3:**
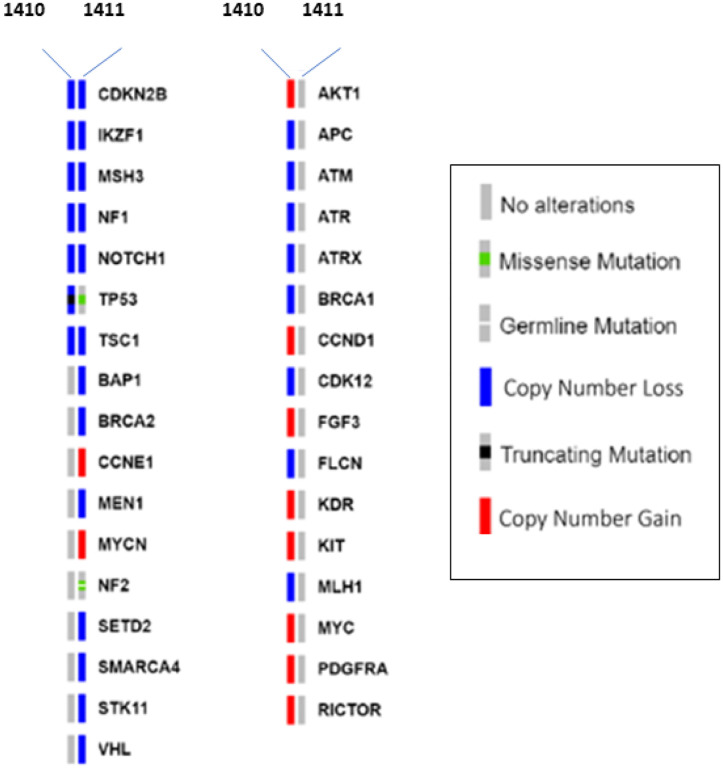
Somatic Mutations Detected by SearchLight DNA in Tumors from Dogs 1411 and 1410. Mutations that are shared by both dogs are shown followed by those detected only in 1411 (bottom of left column) or 1410 (right column). These mutations represent primarily somatic SNVs and CNVs detected in 120 genes via the SearchLight DNA panel based on sequencing matched tumor and normal tissue for each dog. One germline sequence variant was detected in NF2 in Dog 1411, but was not shared by Dog 1410. Six genes bore similar somatic mutations in both cases whereas 27 genes bore mutations in only a single sibling’s tumor.

Mean target sequencing coverage averaged 313x across both dogs’ tumor and normal samples. Few single nucleotide variations (SNV)s were detected (Fig. 3). After filtering out known, common, benign single nucleotide polymorphisms (SNPs), 4 SNVs were detected in all four samples including NF2 Glu231Lys in the 1411 tumor and germline DNA (at a 50% and 44% variant allele fraction (VAF), respectively), a somatic TP53 Ser293Phe in the 1411 (elite) tumor only (93% VAF), and a somatic TP53 Cys207fs (82% VAF) in the 1410 (poor) tumor only.

No shared mutations were detected between siblings and no pathogenic germline SNV was detected in either dog. Although an NF2 mutation was observed in the germline and tumor of 1411, this variant is likely benign. The NF2 variant is a known SNP that, though rare in the general canine population (0.09% frequency among 1,172 measured dogs^46^), has not previously been reported in the human or canine cancer literature. The mutation also occurs at a 44–50% VAF, consistent with a heterozygous state with no sign of a second hit in the tumor.

When considering copy number variations (CNVs) that were detected with a log2 fold-change equivalent to at least a single copy gain or loss (≤ −0.35 for a loss and ≥ 0.35 for a gain), 38 genes were found to be impacted by CNVs in both tumors. No CNVs were detected at significant levels in germline samples. Thus, no obvious shared germline pathogenic CNV was observed in SearchLight DNA regions. CNVs detected in the tumors were mostly unique to each sample and demonstrate notable differences in copy number alterations between the two dogs involving genes implicated in osteosarcoma. Both dogs’ tumors exhibited a homozygous CDKN2B loss and partial loss of IKZF1, MSH3, NF1, NOTCH1 and TSC1, 1411 (elite) demonstrated partial losses of BAP1, BRCA2, MEN1, SETD2, SMARCA4, STK11, and VHL, with gains of CCNE1 and MYCN. In contrast, 1410 (poor) demonstrated partial losses in TP53, APC, ATM, ATR, ATRX, BRCA1, CDK12, FLCN, and MLH1, with small gains in RICTOR, AKT1, CCND1, and FGF3, and a more significant gain of chr13 that spanned MYC, KIT, KDR, and PDGFRA.

**Figure 4:**
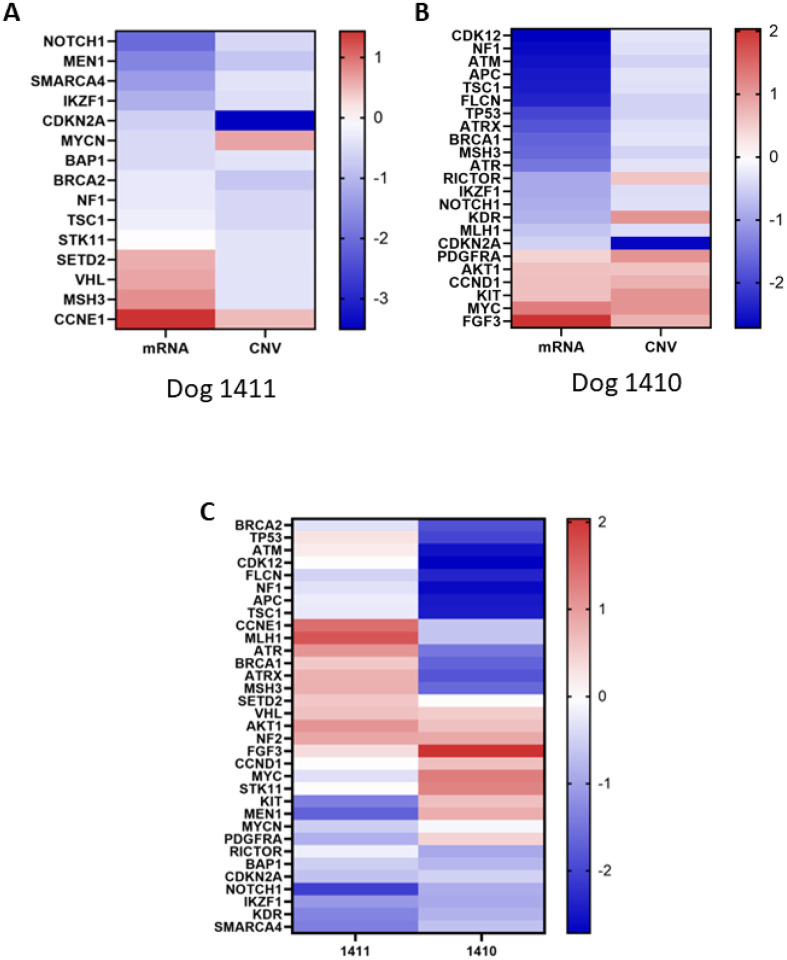
Copy number and gene expression relationships in tumor tissue from dogs 1410 and 1411. A. mRNA expression vs log 2-fold change copy number variation for patient 1411 (Spearman correlation 0.4, p=0.14) B. mRNA expression vs log 2-fold change copy number variation for patient 1410 (Spearman correlation 0.54, p=0.0083). C. mRNA expression for both 1411 and 1410 of 33 genes exhibiting a copy number variation in either dog (Spearman correlation −0.0137, p=0.94).

**Figure 5:**
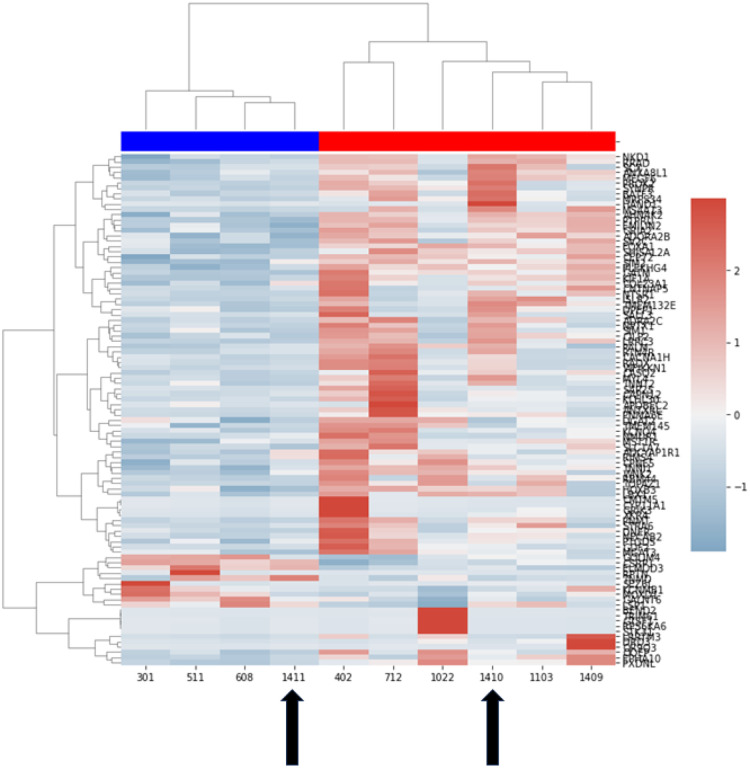
Differentially expressed genes (DEGs) between Rottweilers with disparate clinical outcomes. Ward Clusters of 10 Rottweilers in the DOG^2^ Cohort over 97 differentially expressed genes between Elite responders (Blue) and Poor Responders (Red). Patient ID numbers are listed along the x axis. Dogs 1410 and 1411 are indicated by arrows. A complete gene list is also provided in Supplemental Table 2.

Utilizing the bulk mRNAseq and paired clinical data from n = 10 Rottweiler dogs within the DOG^2^ cohort, which included both 1411 and 1410, correlations between log2 fold changes in CNV and gene expression were explored for specific genes to establish a gene dose-gene expression relationship. In dog 1410 (poor), a significant correlation was seen but not for dog 1411 (elite), likely due to the higher incidence of CNV in 1410 (Fig. 4). We then sought to determine if transcriptionally-defined clusters and/or differentially expressed genes (DEGs) could be identified to define differences between 1410 and 1411, and how they relate to other Rottweilers with osteosarcoma based on known clinical outcomes with equivalent standardized therapy. Our a *priori* definition of elite (DFI > 200 days) and poor (DFI < 200 days) responder groups allowed segregation of dogs and a supervised analysis of DEGs between these two groups (Fig. 5). Although the sample size is small, 97 DEGs (**Supplemental Table 2**), were identified that define these two outcome-linked groups of Rottweilers. We then went on to apply a transcriptional signature originally derived from an external canine osteosarcoma dataset, GS-1, that consists mainly of genes involved in immune responses.^5,28^ This analysis indicates that Rottweilers in the DOG^2^ cohort, including both siblings, appear to have under-expression of GS-1 genes (**Supplemental Fig. 1A**), consistent with an ‘immune low’ environment, which has been previously shown to correlate with immune cell infiltration as demonstrated by labeling of immune cells including macrophages (**Supplemental Fig. 1B**). Although decreased GS-1 enrichment has been associated with poor prognosis^28^, the DFI and survival of the Rottweiler cohort was not significantly different than the remainder of the COTC021/022 cohort (**Supplemental Fig. 2**).

## Discussion

Osteosarcoma is uncommon among humans, and even rarer between siblings, however, studying such a phenomenon can provide key insight into the genetic behavior and pathogenesis of the disease.^47^ As of 2021, 48 case reports describe 42 human siblings from 19 families and 3 patients who had an unaffected identical twin, with the first occurrence dating back to 1930.^48–50^ Instances of osteosarcoma between children and parents or between cousins have been recorded as well.^51,52^ While the etiology of osteosarcoma is largely unknown, research suggests heritable components may contribute to tumor development in both canines and humans.^53–56^ In human osteosarcoma patients, this includes Paget’s disease, Li-Fraumeni syndrome, hereditary retinoblastoma, ATR-X syndrome, Rothmund-Thomson syndrome, and Werner and Bloom syndromes. In dogs, germline variants in APC2, BLM, BRCA2, TP53, RB1, WRN, and CDKN2B have also been observed.^5^ Although not evident in the sibling dogs described herein, genomic sequencing studies of related dogs may provide insight into germline variants in genes not previously linked to OS, as described by Mirabello et al in a study of over 1200 patients with osteosarcoma.^56^

The goals of this case report were to describe the clinical, histologic and molecular/genomic features Rottweilers within the DOG^2^ cohort and specifically, a sibling pair of affected Rottweiler dogs with disparate outcomes with similar therapy. Review of medical records and associated clinical trial data did not identify obvious differences within their home environment, histologic or clinical features, or therapeutic management. This directed us to examine the molecular features of their tumors. What we describe here is the clinical and transcriptomic landscape of Rottweiler dogs from a prospective, randomized clinical trial with evidence of a differential genomic and transcriptional program between 2 sibling Rottweiler dogs with contemporaneously occurring osteosarcoma but vastly different clinical outcomes.

In this study, we used SearchLight to investigate CNV changes that relate to genes with known and/or suspected association with osteosarcoma in prior canine and human literature. SearchLight DNA is a commercially-available pan-cancer tumor genomic sequencing panel that reports on multiple mutation types, including single-nucleotide variants, copy number variants, and internal tandem duplications in 120 pre-selected cancer genes. The assay covers 1,358 exonic regions and 429 exon-proximal regions of the genome across 11,554 probes targeting 482.3 kbp of sequence space and was curated based upon prior experience with canine tumors, content of comparable human gene panels, and review of canine and human cancer genomic literature.^33, 57–59^ Although the data from these sibling Rottweiler dogs did not uncover a shared germline pathogenic variant to explain their contemporaneous osteosarcoma development, we were able to describe genomic changes in their respective osteosarcomas that may have a role in progression or resistance to therapy.

The SearchLight results demonstrated CNV changes occurring in both dogs that relate to genes with known and/or suspected association with osteosarcoma in prior canine and human literature. Both dogs had shared copy number losses in a subset of genes, such as CDKN2B and NOTCH1, but the remainder of alterations appeared mutually exclusive to each dog’s tumor. For example, in dog 1411, a segmental gain in CFA13 was observed that includes KDR, KIT, and PDGFRA. An analogous segmental amplification of human chromosome 4q11–12 involving KIT, KDR and PDGFRA has been identified in 6–20% of osteosarcoma patients.^60^ This gain has been implicated as a both druggable event and a negative prognostic factor in some human cancers.^61^ Dog 1411 also demonstrated a gain in MYC, also located on CFA 13. MYC amplification has garnered much attention recently as a potentially prognostic biomarker for human OS.^62^ Larger studies of this gene-dense region are needed to define an association between segmental and/or single-gene amplification and prognosis in canine osteosarcoma.

The SearchLight assay provided data on TP53, a known driver of osteosarcoma in both humans and dogs. Homologous loss-of-function mutations in TP53 were observed, with dog 1410 exhibiting both a copy number loss and truncating mutation, and dog 1411 exhibiting a missense mutation. Alterations in TP53 are the most common genomic lesions observed in osteosarcomas of both dogs and humans, but the nature of the alterations varies. In dogs, point mutations appear to dominate while in humans, both point mutations and structural variations, particularly translocations involving intron 1, are seen.^63,64^ As computational tools become more widely available for assessment of structural variations in canine genomes, more data will emerge to characterize these alterations more fully.

Losses of CDKN2A, BRCA2, SETD2, ATRX and others have also been identified in cohorts of human osteosarcoma patients.^65,66^ Both dogs’ tumors exhibit a homozygous CDKN2B loss but no evidence of a germline event at this locus. Both CDKN2A and CDKN2B act as tumor suppressors through encoding proteins p16^INK4a^, p14^ARF^ and p15^INK4b^, which regulate G_1_ cell cycle arrest.^66^ To this point, genome-wide association studies (GWAS) carried out in 3 high-risk breeds (Greyhounds, Rottweilers, and Irish Wolfhounds) as well as the Leonberger dog, implicated regulatory elements upstream of the CDKN2A/B locus as highly associated with osteosarcoma development and possibly responsible for disruption of enhancer elements and thus altered expression of genes responsible for cell cycle control in this region.^25,67^ It is possible that identification of alterations upstream of the CDKN2A/B locus can be identified through whole-genome sequencing of both tumor and normal tissues from dogs 1410 and 1410, as well as other Rottweilers in the DOG^2^ cohort. A recent GWAS study in Bernese Mountain Dogs, Rottweilers, golden retrievers and flat-coated retrievers across 3 hematopoietic cancers (histiocytic sarcoma, lymphoma and mast cell tumor) also implicates the CDKN2A locus as well as other loci on canine chromosomes 5 and 20.^68^

The histone methyltransferase SETD2 is a tumor-suppressor gene that has been documented to harbor mutations in a small subset of human osteosarcomas.^65^ In a study of whole-exome sequencing in 66 dogs with osteosarcoma including 21 Rottweilers, SETD2 was the second most frequently mutated gene after TP53; this was further supported by a second sequencing study which reported SETD2 mutations in 42% of 24 canine patients.^5,69^ Dog 1411 had copy number loss of this gene in their tumor based on the SearchLight assay. SETD2 has been implicated as a potential driver in both canine and human osteosarcoma, which is consistent with data that implicates epigenetic modulation in tumor progression and differential outcomes.^70^ Additional work is needed to define the specific role of SETD2 in gene regulation and the DNA damage response (DDR), which would be highly relevant in a cancer type that is characterized by treatment with DNA damaging agents such as platinum chemotherapy. This could be further exacerbated by loss of or mutations in DDR genes such as ATM, ATR, ATRX, BRCA1/2 and BAP1.^71^ As compared with the genomes of other pediatric cancers, human osteosarcoma genomes have a relatively high-level, homologous recombination–deficient signature (typically characteristic of *BRCA1*/*2*-deficient cancers).^71,72^ It has been suggested that alterations in these genes in osteosarcoma may be best identified through copy number variation and not through whole-genome or whole-exome sequencing^10,72^, which is a current focus of work within the larger DOG^2^ cohort. SETD2 loss in dog 1411 may also be associated with sirolimus exposure and response to mTOR inhibition. Although the pharmacokinetic profile for this dog suggests inadequate exposure to the drug, it is possible that some measure of mTOR inhibition may have occurred within tumor tissue, which may have been augmented by the SETD2 deficient nature of 1411’s primary tumor. SETD2 loss or inactivation has been associated with enhanced response to mTOR inhibition through alterations in oxidative metabolism and protein synthesis pathways.^73,74^

Shared loss of NOTCH1 in both dogs also carries relevance to osteosarcoma, as the NOTCH signaling pathway plays an important role in osteogenic differentiation.^75^ Dysregulation of this pathway is linked to occurrence and progression of defects involving this process.^76^ The functional status or expression level of NOTCH pathway receptors and target genes has been associated with mixed impacts on proliferation, apoptosis, and clinical variables in both human and canine osteosarcoma.^77–79^ This may be due to the time-sensitive expression of NOTCH receptors during osteogenic differentiation as some members (NOTCH1, NOTCH3) maintain the undifferentiated state of osteoprogenitor cells, while others (NOTCH2, NOTCH4) promote osteoblastic differentiation.^80^

In both dogs, analysis of the bulk mRNAseq data and its relationship to copy number data for the genes contained within the SearchLight panel provided the opportunity to link gene expression to gene dose. Although many factors can influence gene expression aside from loss or gain of copies of individual genes, this data demonstrates a significant relationship between CNV and mRNAseq of selected genes from dog 1411 (elite). Whole-genome sequencing data could be assessed alongside the bulk mRNAseq data from these and other dogs for which paired data are available within the DOG^2^ cohort, to make additional observations on the relationship between these two complementary datasets.

The dataset and case reports presented herein provides interesting insight into differential genomic lesions that may influence outcomes and allowed comparison of these two sibling dogs to other non-related Rottweilers within the larger DOG^2^ cohort. Our study did not uncover a shared germline pathogenic variant which could help explain the development of osteosarcoma in related dogs. The exploration of cancer susceptibility is best performed in the context of a genome-wide association (GWAS) study. In contrast to humans, osteosarcoma in dogs is generally thought to be highly heritable with some large and giant breed dogs, including Rottweilers, at > 10x fold risk of developing the disease. However, given the high incidence across companion dogs in general and those of mixed breeding, the incidence of canine osteosarcoma cannot be purely explained by heritable risk. Future genomic sequencing studies of both related and unrelated dogs should be prioritized to provide insight into germline variants in genes not previously linked to osteosarcoma in canine and human patients. A better understanding of affected genes and their respective pathways will facilitate the development of diagnostic and therapeutic biomarkers and identification of novel therapeutic targets for osteosarcoma patients.

## Data Availability

mRNAseq datasets referenced in this manuscript are available at GEO, accession number GSE238110; https://www.ncbi.nlm.nih.gov/geo/query/acc.cgi?acc=GSE238110
